# Development of a panel of seven duplex real-time PCR assays for detecting 13 streptococcal superantigens

**DOI:** 10.1186/1476-0711-12-18

**Published:** 2013-07-30

**Authors:** Peng Yang, Xiaomin Peng, Shujuan Cui, Junbin Shao, Xuping Zhu, Daitao Zhang, Huijie Liang, Quanyi Wang

**Affiliations:** 1 Institute for Infectious Disease and Endemic Disease Control, Beijing Center for Disease Prevention and Control (CDC), Beijing Research Center for Preventive Medicine; Capital Medical University School of Public Health, No.16 He Pingli Middle Street, Dongcheng District, Beijing 100013, China; 2 Shanghai ZJ Biotech Co. Ltd., No. 188 Xinjunhuan Road, Pujiang High-tech Park Caohejing Development area, Shanghai 201114, China

**Keywords:** Streptococcal, Superantigens, Duplex real-time PCR

## Abstract

**Background:**

Streptococcal superantigens (SAgs) are the major virulence factors of infection in humans for group A Streptococcus (GAS) bacteria. A panel consisting of seven duplex real-time PCR assays was developed to simultaneously detect 13 streptococcal SAgs and one internal control which may be important in the control of GAS-mediated diseases.

**Methods:**

Primer and probe sequences were selected based on the highly conserved region from an alignment of nucleotide sequences of the 13 streptococcal SAgs. The reaction conditions of the duplex real-time PCR were optimized and the specificity of the duplex assays was evaluated using SAg positive strains. The limit of detection of the duplex assays was determined by using 10-fold serial dilutions of the DNA of 13 streptococcal SAgs and compared to a conventional polymerase chain reaction (PCR) method for evaluating the duplex assays sensitivity.

**Results:**

Using the duplex assays, we were able to differentiate between 13 SAgs from Streptococcus strains and other non-Streptococcus bacteria without cross-reaction. On the other hand, the limit of detection of the duplex assays was at least one or two log dilutions lower than that of the conventional PCR.

**Conclusions:**

The panel was highly specific (100%) and the limit of detection of these duplex groups was at least ten times lower than that obtained by using a conventional PCR method.

## Background

Group A *Streptococcu*s (GAS) (*Streptococcus pyogenes*) is an important gram-positive bacterial pathogen that can cause both invasive and non-invasive infections in humans. GAS-associated diseases are more common in children than in adults [1,2]. Among the factors involved in the virulence of the pathogen, the M protein and a group of exotoxins known as streptococcal superantigens (SAgs) have received considerable attention. Streptococcal SAgs are thought to contribute to the pathogenesis of severe GAS infections by virtue of their potent immunostimulatory activity [3]. Rapid detection of streptococcal SAgs in clinical samples is therefore important in the efficient control of GAS.

At present, 13 streptococcal SAgs have been identified in GAS to date, including *SpeA, SpeC, SpeG, SpeH, SpeI, SpeJ, SpeL, SpeK, SpeM, Ssa*, *SmeZ* and *SpeB, SpeF* exhibiting the SAg activity [4-7]. 13 conventional polymerase chain reaction (PCR) using 1 primer pair for each SAgs performed in the same volume of reaction mixture and using the standard number of cycles used in previous studies of streptococcal SAgs detection [4]. However, these tests are not suitable for effective, fast, high-throughput routine diagnostic screening because they are labor intensive, the samples are prone to contamination and have relatively lower sensitivity. For large scale detection, a rapid and efficient method for surveillance of streptococcal SAgs in clinical samples is required. The aim of this study was to develop a rapid and effective method to simultaneously detect 13 streptococcal SAgs. A panel consisting of seven groups of a duplex real-time PCR assay system described here provides a diagnostic tool for the fast, effective and reliable detection of the 13 currently known streptococcal SAgs.

## Materials and methods

### Bacterial strains

As positive controls, SAgs from Streptococcus MY1, MY3 and FS1 were used in the detection of *SpeB, SpeC, SpeF, Spe I and SmeZ*, for *SpeA, SpeG, SpeJ* and *Ssa*, and for *SpeH, SpeK, SpeL* and *SpeM*. Other non-streptococcal bacteria strains including *Mycoplasma pneumoniae, Epidemic neisseria meningitides, Streptococcus pneumoniae, Haemophilus influenzae, Staphyloccocus aureus Rosenbach (MRSA), Group B Streptococcus (FT67), Group G Streptococcus (XCN14)* were also collected and used in this study.

### Primer and probe design

Primers and probes for the panel of the duplex real-time PCR assays for the detection of 13 streptococcal SAgs were designed targeting the conserved regions of the thirteen streptococcal SAgs gene respectively using Primer Express 3.0 Software (Applied Biosystems, Foster CA). The BLAST program was used to assess and analyse the integrity of the primers and probes (National Center for Biotechnology Information Web site (http://www.ncbi.nlm.nih.gov/BLAST). These were matched with the conserved gene sequences of 13 corresponding SAgs. The sequence of primers and the probe labeled reporter are listed in Table 1.

**Table 1:** Duplex real-time PCR primers and probes used in the detection of streptococcal SAgs

**Groups**	**Name**	**Sequence (5′-3′)**
1	*SpeA*	Forward: CAAGAGGTATTTGCTCAACAAG
		Reverse: CACATTCTCGTGAGTAACAGG
		Probe: FAM-CCCGATCCAAGCCAACTTCACAGA-BHQ1
	*SpeL*	Forward: GTCAGCACCTTCCTCTTTC
		Reverse: ATCTCCCGTTACCTTCCAG
		Probe: HEX-CGCCTGAGCCGTGAAA-BHQ1
2	*SpeB*	Forward: CGGACGTAACTTCTACCATG
		Reverse: TTTGATGCCTACAACAGCAC
		Probe: FAM-AACCGTTGAAGCCGCC-BHQ1
	*Ssa*	Forward: CGGGAACTTACCTTCAATTTG
		Reverse: CTATAACTGCTATTATTCGGAAGG
		Probe: HEX-TGCTCAGTAACACCTCC-BHQ1
3	*SpeC*	Forward: CGTAACTTTCCAGGAAATTGAC
		Reverse: GCTCATGTTTCCCATCTTTTG
		Probe: FAM-CGATTCTGCCGCTTACA-BHQ1
	*SpeH*	Forward: AGTTCCTGTAAACGTGTGG
		Reverse: CCTGAGCGGTTACTTTCG
		Probe: HEX-AACAACAGCCGCCTATG-BHQ1
4	*SpeG*	Forward: AACAGTTTACTTTACAGGAATTTG
		Reverse: TGTTTACTATCTTTAGTAGCAAGG
		Probe: FAM-AAAGGCTCCCCGATGT-BHQ1
	*SpeF*	Forward: CATTGATACCACGACAGCTCTTG
		Reverse: TAGAAGCAAATCGTGATGGCTATC
		Probe: HEX-AGCCGCTCCAATCTACAACGCAGACGA-BHQ1
5	*SpeM*	Forward: TTCGCTATTAACCAACACAGCATC
		Reverse: TGTTATTCCTTGTGTGTGTATCGC
		Probe: FAM-AAGACACTCTCAGTAG-NF-BHQ1
	*SpeI*	Forward: ATATGATCCAACAGAAGTAAAAGG
		Reverse: CAATCGAACTATTGCCATAAGG
		Probe: HEX-AATGAAGGTCCGCCA-BHQ1
6	*SpeJ*	Forward: TCTACTGGTATGATTTCGTATGCG
		Reverse: TCATGGGTACGGAAGTGTAAAATC
		Probe: FAM-TTGTAGCTTAACGTC-NF-MGB
	*SmeZ*	Forward: GCCAATGATTTTAAAGMTGGAG
		Reverse: TATATGCTGTGACTTTTCCTTTTG
		Probe: HEX-CTGTGTTCTCCGTCCCA-BHQ1
7	*SpeK*	Forward: ACGTATCTGAAAAGACACTCTC
		Reverse: ATACCTTGACTTTGTTATTCCTTG
		Probe: FAM-AGAGCAAGCGATACACA-BHQ1
	*Internal control*	Forward: GGTGTATAACGTGTCAGAGACC
		Reverse: CTTCTCCAACATACTATGCAAC
		Probe: HEX-CCTGTCCACCTTCCTC-BHQ1

### Nucleic acid extraction

Nucleic acid extraction was carried out by mixing 100 μl of each sample and 1 μl of the internal control followed by the addition of 100 μl of nucleic acid extraction solution. This mixture was incubated in boiling water at 100°C for 10 min, then centrifuged at 13,000 rpm for 5 min. Five microlitres of the supernatant were removed and used in the detection.

### Conventional PCR assay

The conventional PCR assay was performed using a cycler (Life Express PCR instruments, Yamato, Japan). The PCR reaction and amplification conditions were set up according to the method of Roger Meisa [4]. Briefly, the PCR was carried out in a final volume of 25 μl. The reaction mixture consisted of 200 nM of each forward and reverse primer, 1 × Enzyme Mix, 400 μM of dNTP Mix, 5 μl DNA extract and ddH_2_O was used to make up the remaining volume. Amplification was carried out separately for each gene, *SpeA to -C, SpeF to -M*, *SmeZ*, and *Ssa*, using the respective primers. After an initial denaturation step at 94°C for 5 min, amplification was carried out by using 30 cycles at 95°C for 40 s, hybridization for 1 min, and elongation at 72°C for 1 min 30 s, with annealing at 72°C for 5 min. The PCR products were examined using a standardized E-gel electrophoresis system and E-gel 96 with SYBR Safe (Ethrog Biotechnologies, Tel-Hai, Israel).

### Duplex real-time PCR assay

Seven groups of duplex real-time PCR simultaneous amplifications were optimized using the iCycler (Bio-Rad, Hercules, CA,) and the FAM, HEX two channel method using the primers and probes listed in Table 1. The final optimized 40 μl reaction mixture consisted of 200 nM of each forward and reverse primer, 100 nM of TaqMan probe, 1 × Enzyme Mix, 400 μM of dNTP Mix, 5 μl DNA extract and ddH_2_O was used to make up for the volume. The conditions for the amplification of the seven groups were as follows: 2 min at 94°C, followed by 40 cycles of 15 s at 93°C and 60 s at 55°C. A C*t* value between 34 and 36 was considered weakly positive.

### Specificity of the duplex real-time PCR assay

To assess the specificity of the experimental method, the seven groups of duplex real-time PCR reactions were tested for cross-reactivity among the SAgs positive GAS strains and other closely related non-GAS strains, as listed in Materials and Methods. The results were analyzed based on the specific amplification curve either in the FAM or HEX channel depending on the predetermined amplification parameters.

### Detection limit of the duplex real-time PCR assay

To assess the detection limit of the experimental method, DNA templates of 13 streptococcal SAgs were serially diluted 10-fold ranging from undiluted to 10^-4^. Five different DNA dilutions corresponding to different SAgs were analysed simultaneously using the duplex real-time PCR assay and the conventional PCR assay. The results were analyzed based on the limit of detection the two tests to illustrate the relative sensitivity of each method.

## Results

### Specificity of the duplex real-time PCR assay

To assess the specificity, the Sag positive GAS strains and other closely related non-GAS strains were selected for amplification using the real-time PCR assay. No nonspecific reactions nor any internal cross-amplification was observed (data not shown).

### Detection limit of the duplex real-time PCR assay

To determine the detection limit of the experimental method, the results obtained using the duplex system for the detection of streptococcal SAgs were analyzed and compared to the results obtained from the conventional PCR assay. The DNA was diluted using a 10-fold series ranging from undiluted to 10^-4^. The average limit of detection using the duplex system was at least a 10^-3^ dilution from the undiluted sample and that of the conventional PCR system was at most a 10^-2^ dilution. The detection limit of the duplex real-time PCR assays for the detection of *SpeA*, *SpeC, SpeG* and *SpeM* were observed to be lower by one log unit compared with that observed using the conventional PCR assay. The detection limit of the other nine SAgs was observed to be at least two log units lower than that of the conventional PCR assay. The results showing the detection limits of 13 SAgs are listed in Table 2.

**Table 2:** Comparison of the detection limits of a dilution series of DNA (after amplification) using conventional PCR and duplex real-time PCR*

**Groups**	**Dilution**	**Detection limit**			
		**Conventional PCR**	**Duplex real-time PCR**	**Conventional PCR**	**Duplex real-time PCR**
		*SpeA*		*SpeL*	
**1**	10^0^	+	+	+	+
	10^-1^	+	+	+	+
	10^-2^	+	+	+	+
	10^-3^	+	+	-	+
	10^-4^	-	+	-	+
		*SpeB*		*Ssa*	
**2**	10^0^	+	+	+	+
	10^-1^	+	+	+	+
	10^-2^	-	+	+	+
	10^-3^	-	+	-	+
	10^-4^	-	-	-	+
		*SpeC*		*SpeH*	
**3**	10^0^	+	+	+	+
	10^-1^	+	+	+	+
	10^-2^	+	+	-	+
	10^-3^	-	+	-	+
	10^-4^	-	-	-	-
		*SpeG*		*SpeF*	
**4**	10^0^	+	+	+	+
	10^-1^	+	+	+	+
	10^-2^	+	+	+	+
	10^-3^	-	+	-	+
	10^-4^	-	-	-	+
		*SpeM*		*SpeI*	
**5**	10^0^	+	+	+	+
	10^-1^	+	+	+	+
	10^-2^	+	+	-	+
	10^-3^	-	+	-	+
	10^-4^	-	-	-	-
		*SpeJ*		*SmeZ*	
**6**	10^0^	+	+	+	+
	10^-1^	+	+	+	+
	10^-2^	+	+	-	+
	10^-3^	-	+	-	+
	10^-4^	-	+	-	-
		*SpeK*		*Internal control*	
**7**	10^0^	+	+	+	+
	10^-1^	+	+	+	+
	10^-2^	-	+	+	+
	10^-3^	-	+	+	+
	10^-4^	-	-	+	+

* For each 10-fold DNA dilution, the lowest level of detection of the duplex real-time PCR is shown in addition to the three 10-fold dilutions immediately prior to the lowest limit. Each dilution series was analysed in triplicate to determine relative sensitivity.

## Discussion

To the best of our knowledge, this is the first report of the development of a panel of seven groups of a duplex real-time PCR assay for simultaneously detecting 13 streptococcal SAgs. In the design of these real-time PCRs, an alignment of conserved regions of the target pathogens was made with publicly available GenBank sequences. The primer-probe combination was newly used in the present study and did not cross-react with other similar bacteria strains (data not shown). The specificity of the PCR reactions in these experiments using a novel duplex system was very high (100%), and was able to differentiate between different streptococcal SAgs. Furthermore, the detection limit of the duplex real-time PCR was lower by one or two log units than that of the conventional PCR assay. These results indicate that the duplex system described here may be useful in the clinical setting.

There are several advantages of the duplex real-time PCR system compared with the conventional PCR technique, for example, the ease of performance, high specificity and sensitivity, fast turn-around time, a high-throughput capacity, and minimal carry-over contamination [8]. Real-time PCR assays have been widely utilized for early diagnosis of many other bacterial infections [9]. In this study, an internal control was successfully integrated into the assays for accurate interpretation of negative results. The panel of duplex real-time PCR assays could possibly be used as a rapid and efficient diagnostic tool for 13 streptococcal SAgs in the clinical setting.

## Conclusions

The panel of duplex real-time PCR assays described in this report is a simple, specific, sensitive and rapid method for the detection of 13 streptococcal SAgs in clinical samples. This technique has the potential for use in routine diagnostics.

## Competing interests

The authors declare that they have no competing interests.

## Authors’ contributions

PY, XP, SC carried out the evaluation experiments, data collection and organization, statistical analysis and contributed to writing and to the interpretation of the results. JS, XZ, DZ and HL carried out the duplex real-time PCR and the sequence alignment. QW contributed to the design of the study and assisted in the drafting of the manuscript. All authors have read and approved the manuscript.
